# Augmented Fibroblast Growth Factor-23 Secretion in Bone Locally Contributes to Impaired Bone Mineralization in Chronic Kidney Disease in Mice

**DOI:** 10.3389/fendo.2018.00311

**Published:** 2018-06-11

**Authors:** Olena Andrukhova, Christiane Schüler, Claudia Bergow, Alexandra Petric, Reinhold G. Erben

**Affiliations:** Department of Biomedical Sciences, University of Veterinary Medicine Vienna, Vienna, Austria

**Keywords:** fibroblast growth factor-23, chronic kidney disease, bone mineralization, osteocytes, pyrophosphate, alkaline phosphatase

## Abstract

Chronic kidney disease-mineral and bone disorder (CKD-MBD) is a systemic disorder of mineral and bone metabolism caused by CKD. Impaired bone mineralization together with increased bony secretion of fibroblast growth factor-23 (FGF23) are hallmarks of CKD-MBD. We recently showed that FGF23 suppresses the expression of tissue nonspecific alkaline phosphatase (TNAP) in bone cells by a Klotho-independent, FGF receptor-3-mediated signaling axis, leading to the accumulation of the mineralization inhibitor pyrophosphate. Therefore, we hypothesized that excessive FGF23 secretion may locally impair bone mineralization in CKD-MBD. To test this hypothesis, we induced CKD by 5/6 nephrectomy in 3-month-old wild-type (WT) mice and *Fgf23*^−/−^*/*VDR^Δ/Δ^ (*Fgf23*/VDR) compound mutant mice maintained on a diet enriched with calcium, phosphate, and lactose. Eight weeks postsurgery, WT CKD mice were characterized by reduced bone mineral density at the axial and appendicular skeleton, hyperphosphatemia, secondary hyperparathyroidism, increased serum intact Fgf23, and impaired bone mineralization as evidenced by bone histomorphometry. Laser capture microdissection in bone cryosections showed that both osteoblasts and osteocytes contributed to the CKD-induced increase in *Fgf23* mRNA abundance. In line with our hypothesis, osteoblastic and osteocytic activity of alkaline phosphatase was reduced, and bone pyrophosphate concentration was ~2.5-fold higher in CKD mice, relative to Sham controls. In *Fgf23*/VDR compound mice lacking Fgf23, 5/6-Nx induced secondary hyperparathyroidism and bone loss. However, 5/6-Nx failed to suppress TNAP activity, and bone pyrophosphate concentrations remained unchanged in *Fgf23*/VDR CKD mice. Collectively, our data suggest that elevated Fgf23 production in bone contributes to the mineralization defect in CKD-MBD by auto-/paracrine suppression of TNAP and subsequent accumulation of pyrophosphate in bone. Hence, our study has identified a novel mechanism involved in the pathogenesis of CKD-MBD.

## Introduction

The progressive decline in kidney function associated with chronic kidney disease (CKD) leads to complex changes in mineral and bone metabolism. These changes include alterations in circulating biomarkers, metabolic bone disease, and ectopic, especially vascular calcifications. The term chronic kidney disease-mineral and bone disorder (CKD-MBD) has been coined to better illustrate the association between altered mineral and bone metabolism and cardiovascular morbidity in CKD patients (1). CKD-MBD encompasses metabolic bone disease, vascular calcifications, as well as changes in blood biochemistry such as secondary hyperparathyroidism, hyperphosphatemia, lowered levels of the vitamin D hormone 1α,25-dihydroxyvitamin D_3_ [1,25(OH)_2_D_3_], chronic metabolic acidosis, elevated circulating Wnt (Wingless/integrated-1) inhibitors, and increased concentrations of intact fibroblast growth factor-23 (FGF23) (1, 2).

Fibroblast growth factor-23 is a bone-derived hormone, suppressing urinary phosphate reabsorption by a downregulation of apical membrane expression of sodium phosphate co-transporters in proximal renal tubular epithelium (3, 4). Moreover, FGF23 is a strong transcriptional suppressor of proximal tubular 1α-hydroxylase, the key enzyme for 1,25(OH)_2_D_3_ production (3). In distal renal tubules, FGF23 augments renal calcium and sodium reabsorption (5, 6). Only the intact form of FGF23 is biologically active (7). High-affinity binding of FGF23 requires the concomitant presence of FGF receptors (FGFRs) and of the co-receptor αKlotho (Klotho) in target tissues (8, 9).

Increased blood concentrations of intact FGF23 are one of the earliest biomarkers of CKD. The decline in kidney function in CKD patients causes the circulating intact FGF23 levels to rise. In advanced renal failure, FGF23 serum concentrations can reach levels 1,000-fold above the normal range (10, 11). The reason underlying the upregulation of circulating intact FGF23 in CKD patients is still not entirely clear. One component may be reduced renal elimination through impaired glomerular filtration rate (GFR) (12), but lower renal elimination alone is insufficient to explain the pronounced rise in intact FGF23 in CKD patients. Parathyroid hormone (PTH) and extracellular phosphate are known stimulators of FGF23 secretion (13). However, several studies in human CKD patients have shown that the early increase in circulating intact FGF23 occurs independent of hyperphosphatemia and increased PTH (14). It is conceivable that the CKD-induced increase in circulating pro-inflammatory cytokines may play an important role in causing the rise in FGF23 in patients with early CKD (15, 16).

In bone, the combination of secondary hyperparathyroidism, low 1,25(OH)_2_D_3_, hyperphosphatemia, and metabolic acidosis leads to a syndrome named renal osteodystrophy. The hallmarks of renal osteodystrophy are altered bone turnover (high or low) and impaired bone mineralization (1). Bone turnover in CKD patients is thought to be mainly driven by secondary hyperparathyroidism (1, 17). The mechanisms underlying the impaired bone mineralization are less clear, and may involve secondary hyperparathyroidism, metabolic acidosis, increased circulating Wnt inhibitors, or uremic toxins (1). We recently showed that FGF23 not only targets the kidney in an endocrine manner but also acts as an auto-/paracrine regulator of bone mineralization by suppressing tissue nonspecific alkaline phosphatase (TNAP) expression in osteoblasts and osteocytes (18). One of the substrates of TNAP in bone is pyrophosphate, which is produced by osteoblasts and osteocytes for the regulation of bone mineralization. Pyrophosphate is a potent inhibitor of mineralization by binding to hydroxyapatite crystals (19). In bones of *Hyp* mice, which are characterized by profoundly increased endogenous bony production of Fgf23, we found suppressed osteocytic TNAP activity and accumulation of pyrophosphate (20). Both effects could be rescued by bone-specific ablation of *Fgf23* (20). This finding led us to hypothesize that the CKD-driven upregulation of bony Fgf23 secretion may also locally contribute to impaired bone mineralization in CKD-MBD by suppressing TNAP, leading to accumulation of pyrophosphate. To test this hypothesis, we induced CKD by 5/6-nephrectomy (5/6-Nx) in wild-type (WT) and *Fgf23* deficient mice, and examined mineral and bone metabolism, 8 weeks after 5/6-Nx.

## Materials and Methods

### Animals

All animal studies were approved by the Ethical Committee of the University of Veterinary Medicine, Vienna and by the Austrian Federal Ministry of Science and Research and were undertaken in strict accordance with prevailing guidelines for animal care (permit No. BMWF-68.205/0054-II/3b/2013). All efforts were made to minimize animal suffering. All experiments were performed on 3-month-old male WT and *Fgf23*^−/−^/VDR^Δ/Δ^ mice on C57BL/6N genetic background. To generate *Fgf23*^−/−^/VDR^Δ/Δ^ mice, VDR^+/Δ^/*Fgf23*^+/−^ double heterozygous mice were mated, and the offspring was genotyped by multiplex PCR using genomic DNA extracted from the tail as described (21, 22). All mice were kept at 24°C with a 12/12 h light/dark cycle, and were allowed free access to tap water and a diet containing 2.0% calcium, 1.25% phosphorus, 20% lactose, and 600 IU vitamin D/kg (Ssniff, Soest, Germany). This diet was shown to normalize mineral homeostasis in vitamin D deficient mice and rats, and in mice lacking a functional VDR (23). Urine was collected in metabolic cages for a 12-h period overnight before necropsy. All mice received subcutaneous calcein double labeling (20 mg/kg, s.c.), 4 and 2 days before necropsy. At necropsy, mice were exsanguinated from the abdominal V. cava under general anesthesia (ketamine/xylazine, 100/6 mg/kg i.p.) for serum collection.

### 5/6-Nephrectomy (5/6-Nx) Model

The 5/6 nephrectomy (5/6 Nx) was performed by a two-stage procedure (24) under isoflurane anesthesia. Pain was managed by s.c. injections of buprenorphine, metamizol, and meloxicam. In addition, the animals received the analgesic piritramid *via* the drinking water for 3 days postsurgery, starting 6 h postsurgery. At the first stage (week 1), the left kidney was exposed *via* a left flank incision, and decapsulated to avoid ureter and adrenal damage. Thereafter, the upper and lower poles were resected. Bleeding was controlled by microfibrillar collagen hemostasis (Gelaspon, Chauvin Ankerpharm, Berlin). The resected upper and lower poles were weighed to ascertain 2/3-resection. One week later, the entire right kidney was removed *via* a right flank incision. In the Sham control mice, the left kidney was exposed *via* a left flank incision at step 1, and the right renal artery was identified after a right flank incision at step 2. After each flank incision, muscles and skin were appropriately repositioned and sewed.

### Serum and Urine Biochemistry

Serum phosphorus and creatinine (Crea) as well as urinary creatinine were analyzed on a Cobas c111 analyzer (Roche). GFR was calculated based on the endogenous creatinine clearance (GFR = Urinary Crea/Serum Crea × Urine volume per min). Serum intact PTH (Immutopics) and serum intact Fgf23 (Kainos) were determined by ELISA.

### Bone Mineral Density (BMD) Measurements

Bone mineral density of the left tibia and L2 lumbar vertebra was measured by peripheral quantitative computed tomography (pQCT) using an XCT Research M+ pQCT machine (Stratec Medizintechnik). The voxel size was 70 µm. One 0.2-mm-thick slice in the tibial shaft at 2 mm proximal to the tibiofibular junction, and three slices in the proximal tibial metaphysis located 1.5, 2, and 2.5 mm distal to the proximal tibial growth plate were measured. In the L2 vertebra, three slices were measured, one in a mid-transversal plane, and two located 0.5 mm rostral and caudal of the mid-transversal plane. BMD values of the tibial metaphysis and L2 lumbar vertebral body were calculated as the mean over three slices. A threshold of 600 mg/cm^3^ were used for calculation of cortical BMD at the tibial shaft, and a threshold of 450 mg/cm^3^ was used for the discrimination between trabecular and cortical BMD in both the tibial metaphysis and the L2 vertebra.

### Bone Histology and Histomorphometry

Proximal tibiae were fixed in 4% paraformaldehyde for 24 h, processed for methylmethacrylate embedding, sectioned at 3 µm thickness using a HM 355S microtome (Microm, Walldorf, Germany), and stained with von Kossa/McNeal and for tartrate resistant acid phosphatase enzyme activity as described (25). Undeplasticized and unstained sections mounted with Fluoromount (Serva) were used for calcein-based measurements. All histomorphometric measurements were made with the help of a semiautomatic system (OsteoMeasure, OsteoMetrics) as described (26).

### TNAP Histochemistry

Histochemical TNAP staining was performed as described previously (20). In brief, deplastified sections were incubated with Vector Red alkaline phosphatase staining kit (Vector Laboratories) and counterstained with DAPI. The sections were analyzed using a Zeiss Axioskop 2 microscope. The fluorescence signal was quantified using Image J. Relative fluorescence of osteoblasts was quantified along the bone surface. For the quantification of relative fluorescence in osteocytes, at least 90 osteocytes per animal were used, and fluorescence was normalized to cell number.

### Pyrophosphate Measurement

Femurs were cut in half at the diaphysis, and the bone marrow was flushed out. Minerals and pyrophosphate were extracted using 300 µL 1.2 M HCl at 4°C overnight under light protection. Subsequently, HCl was evaporated at 99°C within 1–2 h, and samples were re-suspended in 0.5–1 mL of assay buffer. The amount of pyrophosphate was quantified using the PhosphoWorks Fluorimetric Pyrophosphate Assay (AAT Bioquest #21611) according to the manufacturer’s protocol and was normalized to wet weight of the bone.

### Laser Capture Microdissection (LCM)

Distal femurs were snap-frozen in liquid nitrogen with OCT compound (Sakura Finetek, Zoeterwoude, Netherlands). 4-μm-thick cryosections were cut on a cryotome (Leica Kryostat 1720), using the cryotape method as described (27). Cryosections were quickly stained with HistoStain (Arcturus), and cancellous and cortical bone osteoblasts and osteocytes (~100–200 cells per sample each) were harvested using a Veritas (Arcturus) LCM system as described (28).

### RNA Isolation and Quantitative RT-PCR

RNA was extracted from LCM-harvested samples using the PicoPure RNA isolation kit (Thermo Fisher Scientific), and RNA quality was determined using the 2100 Bioanalyzer (Agilent Technologies). After first-strand cDNA synthesis (iScript cDNA Synthesis Kit, Bio-Rad), quantitative RT-PCR was performed on a Rotor-Gene 6000 (Corbett Life Science) using SsoFastTM EvaGreen PCR kit (Bio-Rad). A melting curve analysis was done for all assays. Primer sequences are available on request. Efficiencies were examined by standard curve. Gene expression data were corrected for efficiency and normalized to ornithine decarboxylase antizyme-1 (*Oaz1*) as housekeeping gene.

### Statistical Analyses

The aim of the study was to compare Sham and CKD C57BL/6 mice and Sham and CKD *Fgf23*/VDR mice. The investigators were not blinded as to the type of intervention. We used 10–12 mice per group. For some parameters, data from two identical experiments were pooled. The power analysis of the study was based on our experience with similarly designed mouse experiments, which have shown that the SD of total proximal tibial BMD is about 35 mg/cm^3^ in 4- to 5-month-old male C57BL/6 mice. Based on this SD, a group size of 10–12 animals is sufficient to detect a difference of about 45 mg/cm^3^ total BMD (corresponding to an about 10% change in total BMD) with a power β = 80% and an error α = 5%. Statistics were computed using Prism 7.03 (GraphPad Software Inc.). The data from two groups (Sham and CKD) were analyzed by unpaired *t*-test. Equality of variances was tested by *F*-test. In case the variances were not equal, the data were analyzed using the non-parametric Mann–Whitney *U*-test. When more than two groups were compared, the data were analyzed by one-way analysis of variance followed by Holm–Sidak’s multiple comparisons test. *P* values of less than 0.05 were considered significant. The data are presented as the mean ± SEM.

## Results

### 5/6-Nephrectomy-Induced CKD Is Associated With Increased Osteoblastic and Osteocytic Fgf23 Secretion

**Figure 1:**
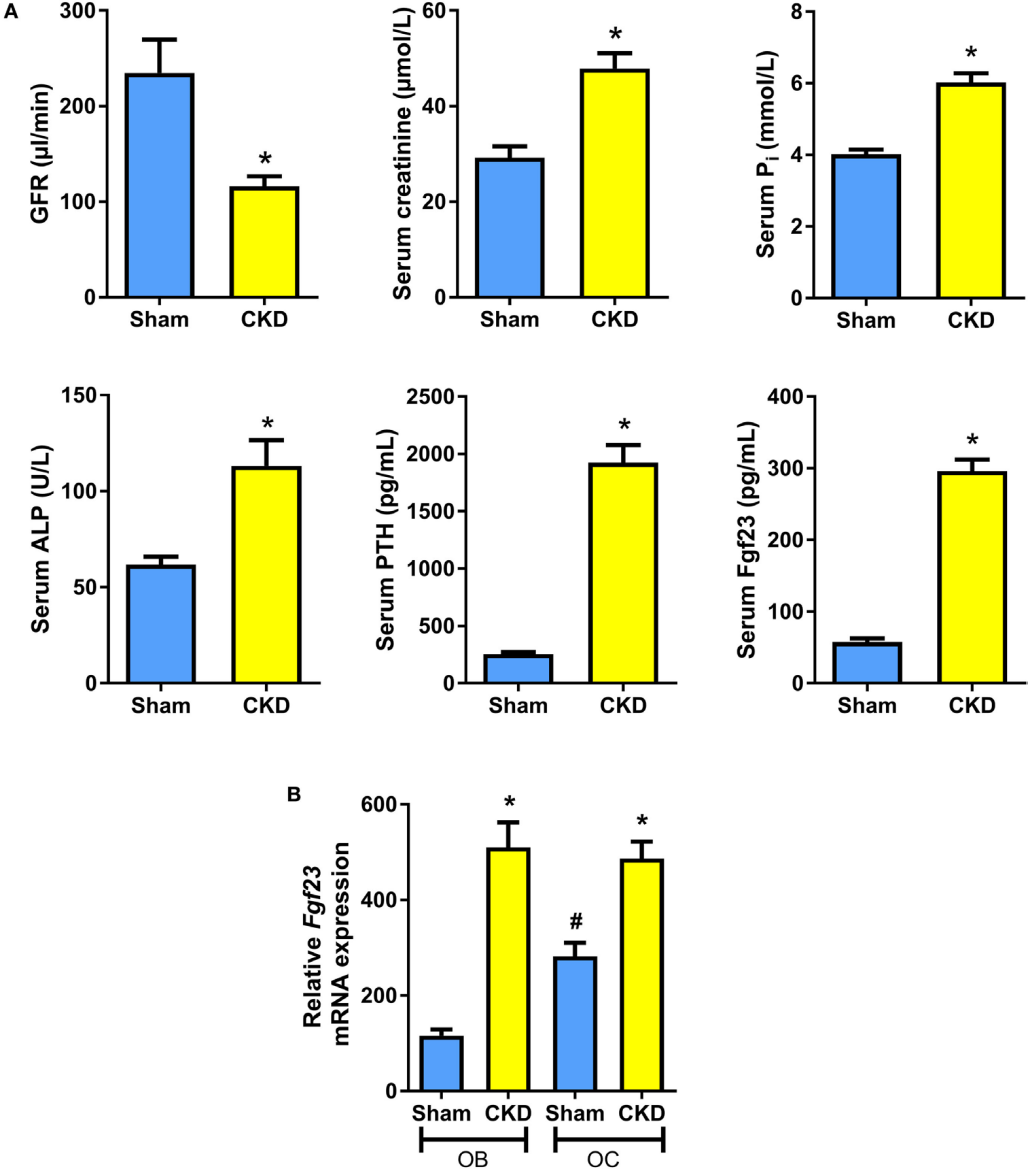
5/6-nephrectomy induces chronic kidney disease (CKD) and increased osteoblastic and osteocytic *Fgf23* mRNA expression in mice on high phosphate diet. **(A)** Glomerular filtration rate (GFR) (*n* = 12–13 each), serum creatinine (*n* = 12 each), serum phosphate (*n* = 12 each), serum alkaline phosphatase (ALP) activity (14 Sham, 20 CKD), serum parathyroid hormone (PTH) (*n* = 12 each), and serum intact Fgf23 (*n* = 5 each) in 5-month-old male C57BL/6 Sham and CKD mice on the phosphate-rich rescue diet, 8 weeks postsurgery. *P < 0.05 vs. Sham by *t*-test or Mann–Whitney *U*-test as appropriate. **(B)** *In situ* mRNA expression profiling of osteoblasts (OB) and osteocytes (OC) harvested by laser capture microdissection in 4-μm-thick distal femoral cryosections from 5-month-old male C57BL/6 Sham (*n* = 7–8) and CKD (*n* = 2) mice on rescue diet, 8 weeks postsurgery. *P < 0.05 vs. Sham, ^#^*P* < 0.05 vs. Sham OB by one-way analysis of variance followed by Holm–Sidak’s multiple comparisons test. Data in **(A,B)** are mean ± SEM.

It is known that C57BL/6 mice are relatively resistant against the development of CKD after 5/6 nephrectomy (5/6-Nx), probably due to low renin activity in this strain of mice (24, 29, 30). Therefore, all mice were maintained on a diet enriched with calcium, phosphate, and lactose. This diet was named rescue diet because it normalizes mineral homeostasis in mice with deficient vitamin D signaling (23). It is well known that phosphate-rich diets accelerate disease progression in 5/6-Nx mice (31). In the rescue diet, lactose stimulates intestinal calcium and phosphate uptake by a vitamin D-independent, paracellular mechanism (32). Eight weeks after 5/6-Nx, GFR as measured by endogenous creatinine clearance was reduced by about 60% relative to Sham controls (Figure 1A). Serum creatinine was distinctly increased in 5/6-Nx mice (Figure 1A). Furthermore, 5/6-Nx mice showed hyperphosphatemia, increased total serum alkaline phosphatase (ALP) activity, severe secondary hyperparathyroidism, and elevated serum intact Fgf23 levels, relative to Sham controls (Figure 1A). Collectively, these data demonstrate that 5/6-Nx mice on a phosphate-rich diet develop CKD within 8 weeks postsurgery, which corresponds in severity approximately to stage 3 of human CKD (2).

It is still controversial whether osteoblasts or osteocytes are the major Fgf23-producing cells in CKD. To shed more light on this issue, we harvested osteoblasts and osteocytes in bone cryosections of Sham and CKD mice, employing LCM, a recently established technology (28). In Sham mice, *Fgf23* mRNA expression in osteocytes was about threefold higher compared with osteoblasts (Figure 1B). CKD induced an upregulation of *Fgf23* mRNA abundance in both osteoblasts and osteocytes (Figure 1B). Although it is clear that Fgf23 protein secretion may not be directly related to changes in *Fgf23* mRNA expression, our findings suggest that both cell types are involved in the CKD-driven increase in bony Fgf23 production.

### CKD Mice Are Characterized by Osteopenia and Impaired Bone Mineralization

**Figure 2:**
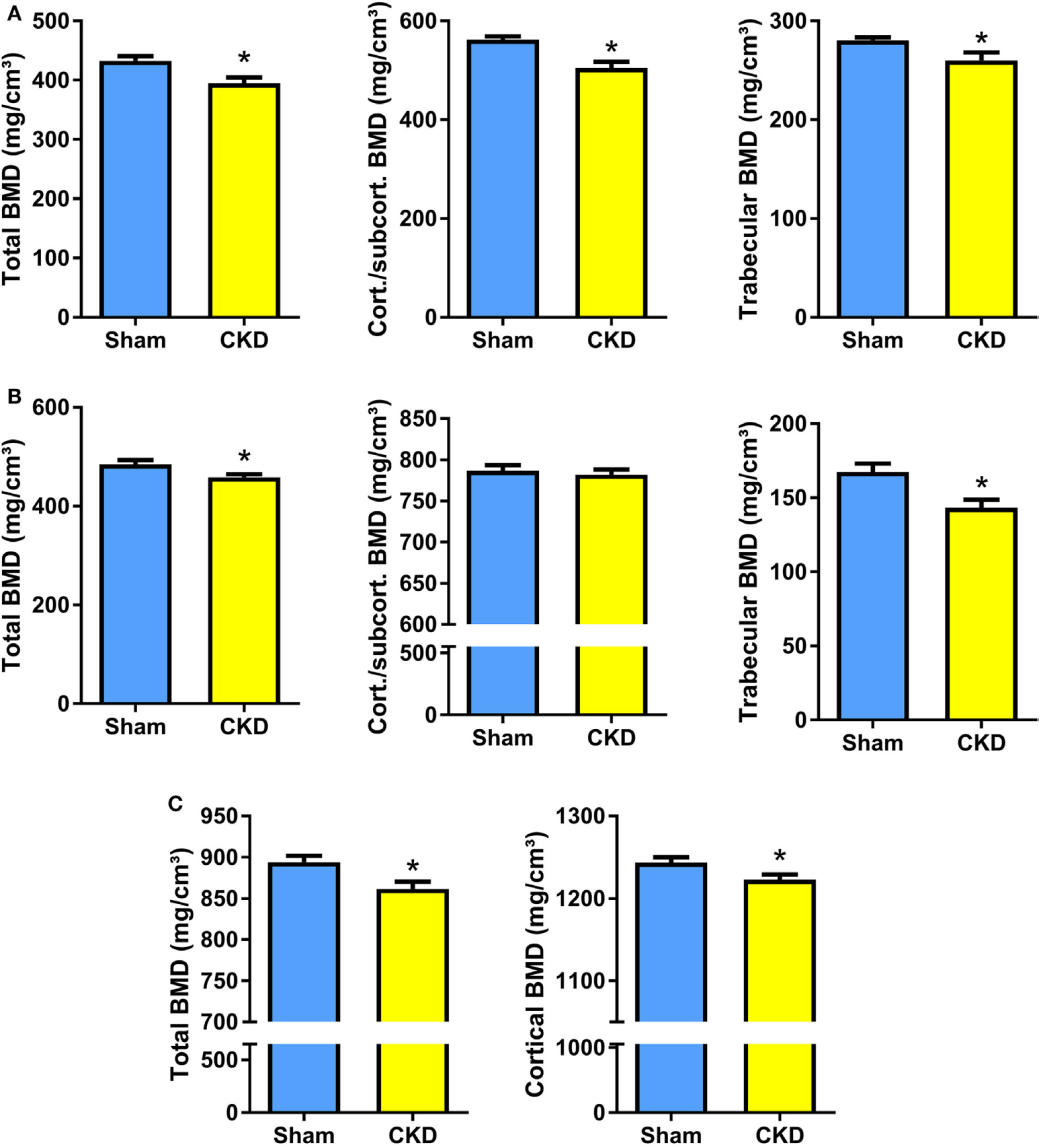
Chronic kidney disease (CKD) induces bone loss in the axial and appendicular skeleton. **(A–C)** Total, cortical/subcortical, and trabecular bone mineral density (BMD) of the L2 lumbar vertebra (17 Sham, 7–9 CKD for each parameter) **(A)** and of the proximal tibial metaphysis (*n* = 16–17 each) **(B)**, as well as total and cortical BMD of the tibial shaft (*n* = 17 each) **(C)** in 5-month-old male C57BL/6 Sham and CKD mice on rescue diet, 8 weeks postsurgery. Data in **(A–C)** are mean ± SEM. *P < 0.05 vs. Sham by *t*-test or Mann–Whitney *U*-test as appropriate.

**Figure 3:**
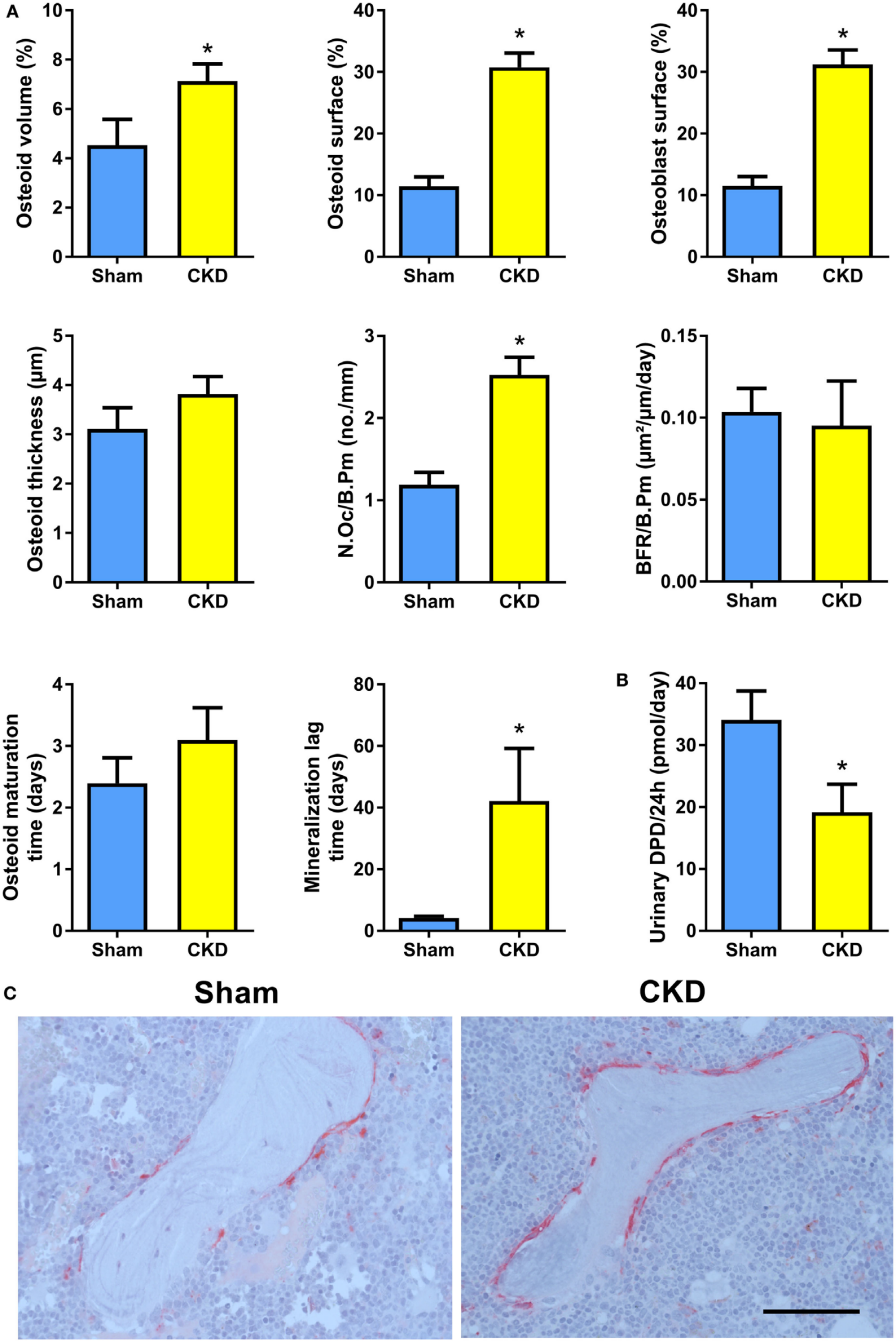
Chronic kidney disease (CKD) mice are characterized by impaired bone mineralization. **(A)** Osteoid volume (14 Sham, 24 CKD), osteoid surface (14 Sham, 24 CKD), osteoblast surface (14 Sham, 24 CKD), osteoid thickness (14 Sham, 24 CKD), osteoclast number per bone perimeter (N.Oc/B.Pm) (14 Sham, 24 CKD), bone formation rate per bone perimeter (BFR/B.Pm) (13 Sham, 8 CKD), osteoid maturation time (13 Sham, 8 CKD), and mineralization lag time (13 Sham, 8 CKD) measured by histomorphometry in cancellous bone of the proximal tibial metaphysis in 5-month-old male C57BL/6 Sham and CKD mice on rescue diet, 8 weeks postsurgery. *P < 0.05 vs. Sham by *t*-test or Mann–Whitney *U*-test as appropriate. **(B)** 24-h urinary excretion of deoxypyridinoline (DPD) (*n* = 16–17 each) in 5-month-old male C57BL/6 Sham and CKD mice on rescue diet, 8 weeks postsurgery. *P < 0.05 vs. Sham by *t*-test. Data in **(A,B)** are mean ± SEM. **(C)** Representative images of tartrate resistant acid phosphatase (TRACP)-stained proximal tibial sections of Sham and CKD mice, showing increased numbers of TRACP-positive osteoclasts in CKD mice. Bar = 100 µm in **(C)**.

In agreement with the well-known fact that the CKD is associated with metabolic bone disease, CKD mice in our study were characterized by small, but significant reductions in total, cortical/subcortical, and trabecular BMD at the spine and at the proximal tibial metaphysis compared with Sham mice (Figures 2A,B). In addition, total and cortical BMD at the tibial shaft were lower in CKD mice, relative to Sham controls (Figure 2C). Cancellous bone histomorphometry showed that the CKD-induced osteopenia was associated with distinctly increased osteoid volume, osteoid surface, osteoblast surface, and osteoclast numbers (Figures 3A,C). Overt signs of impaired bone mineralization were absent in CKD mice, as evidenced by unchanged osteoid thickness, osteoid maturation time, and bone formation rate (Figure 3A). However, CKD mice showed a ~10-fold increase in mineralization lag time (Figure 3A), relative to Sham controls. The calculation of mineralization lag time is based on the adjusted apposition rate, which includes OFF periods in which osteoblasts are not actively mineralizing (33, 34). The pronounced CKD-induced increase in mineralization lag time is indicative of impaired bone mineralization, because a much smaller percentage of osteoblasts was actively mineralizing in CKD vs. Sham mice. In contrast to the elevated osteoclast numbers in CKD mice (Figures 3A,C), 24-h urinary excretion of collagen crosslinks was found to be reduced in CKD mice (Figure 3B). Hence, bone resorption at the functional, whole body level appeared to be decreased in CKD relative to Sham mice. Taken together, these results indicate that 5/6-Nx-induced bone loss was associated with impaired bone mineralization. However, despite the profound elevation in circulating intact PTH in CKD mice (Figure 1A), clear functional evidence of increased bone turnover, i.e., increased bone formation rate or collagen crosslink excretion, was absent in CKD mice, 8 weeks postsurgery.

### Osteoblastic and Osteocytic TNAP Activity Is Suppressed, and Pyrophosphate Concentrations Are Increased in Bones of CKD Mice

**Figure 4:**
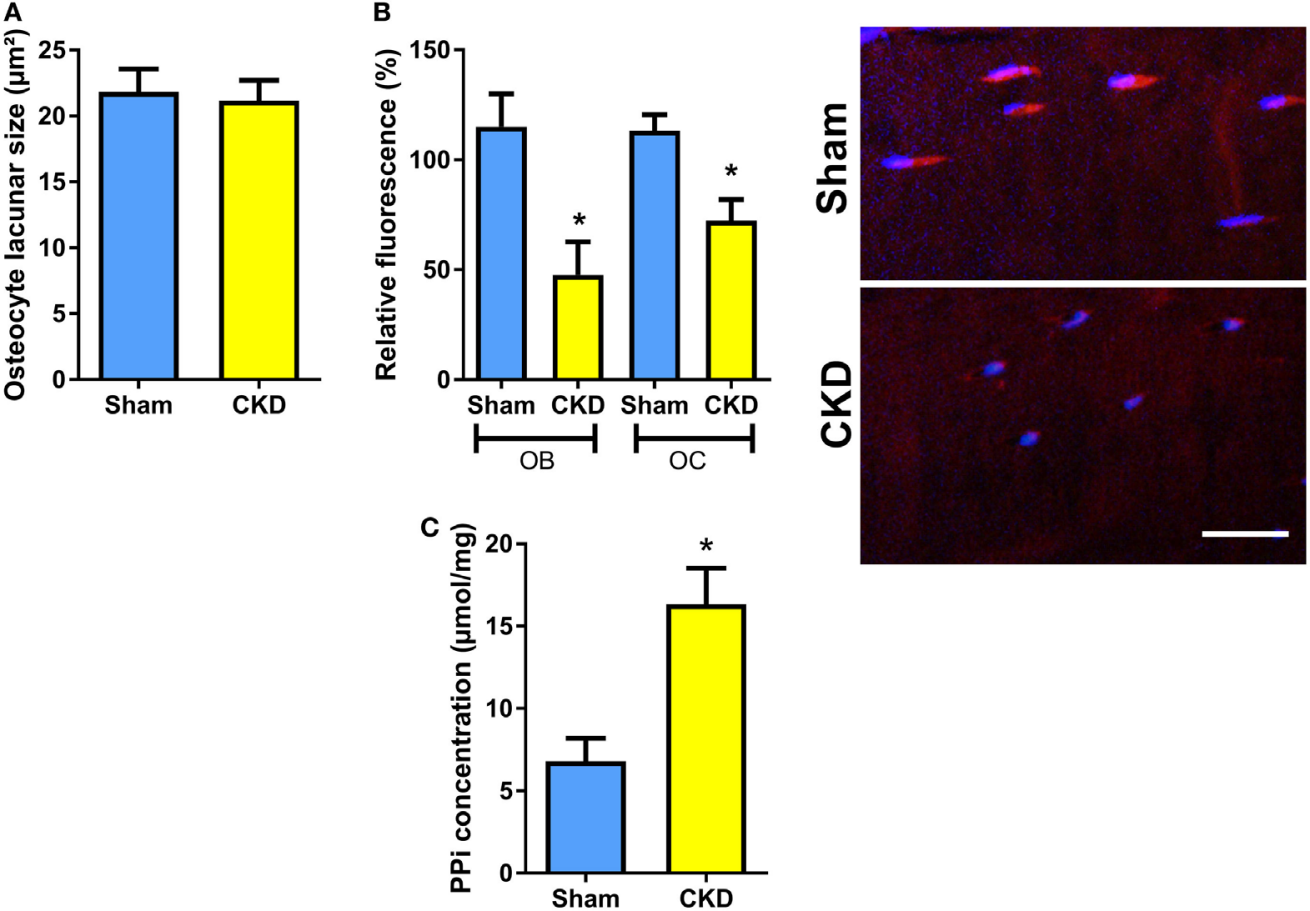
Decreased tissue nonspecific alkaline phosphatase (TNAP) enzyme activity and increased pyrophosphate concentrations in bones of chronic kidney disease (CKD) mice. **(A)** Mean area of osteocyte lacunae measured by histomorphometry in tibial cortical bone (*n* = 13–14 each), **(B)** quantification of relative fluorescence after histochemical TNAP staining of osteoblasts (OB) and osteocytes (OC) in undecalcified sections of proximal tibiae (*n* = 3 each) and representative images of cortical bone osteocytes in Sham and CKD mice, and **(C)** pyrophosphate (PPi) concentration in extracts of femurs (*n* = 3–5 each) from 5-month-old male C57BL/6 Sham and CKD mice on rescue diet, 8 weeks postsurgery. Data in **(A–C)** are mean ± SEM. In **(B)**, * denotes *P* < 0.05 vs. Sham by one-way analysis of variance followed by Holm–Sidak’s multiple comparisons test. In **(C)**, * denotes *P* < 0.05 vs. Sham by *t*-test. Bar = 20 µm in **(B)**.

To test whether the CKD-induced impairment in bone mineralization in cancellous bone would also have implications for mineralization in osteocyte lacunae, we measured osteocyte lacunar size in tibial cortical bone of Sham and CKD mice in von Kossa-stained bone sections. However, osteocyte lacunar size remained unchanged in CKD animals, relative to Sham controls (Figure 4A). We reported earlier that increased local endogenous production of Fgf23 suppresses TNAP activity in osteocytes of *Hyp* mice (20). To examine whether a similar mechanism is also operative in CKD mice, we measured TNAP activity by histochemical analysis in plastic-embedded bone sections. Relative TNAP activity was indeed lower in osteocytes and osteoblasts of CKD mice compared with Sham controls (Figure 4B). The decrease in TNAP activity was associated with increased pyrophosphate concentrations in bones of CKD mice (Figure 4C). These findings support that notion that locally secreted Fgf23 suppresses TNAP in osteocytes, which leads to secondary accumulation of the mineralization inhibitor pyrophosphate.

### Genetic Ablation of Fgf23 Rescues CKD-Induced TNAP Suppression and Pyrophosphate Accumulation

**Figure 5:**
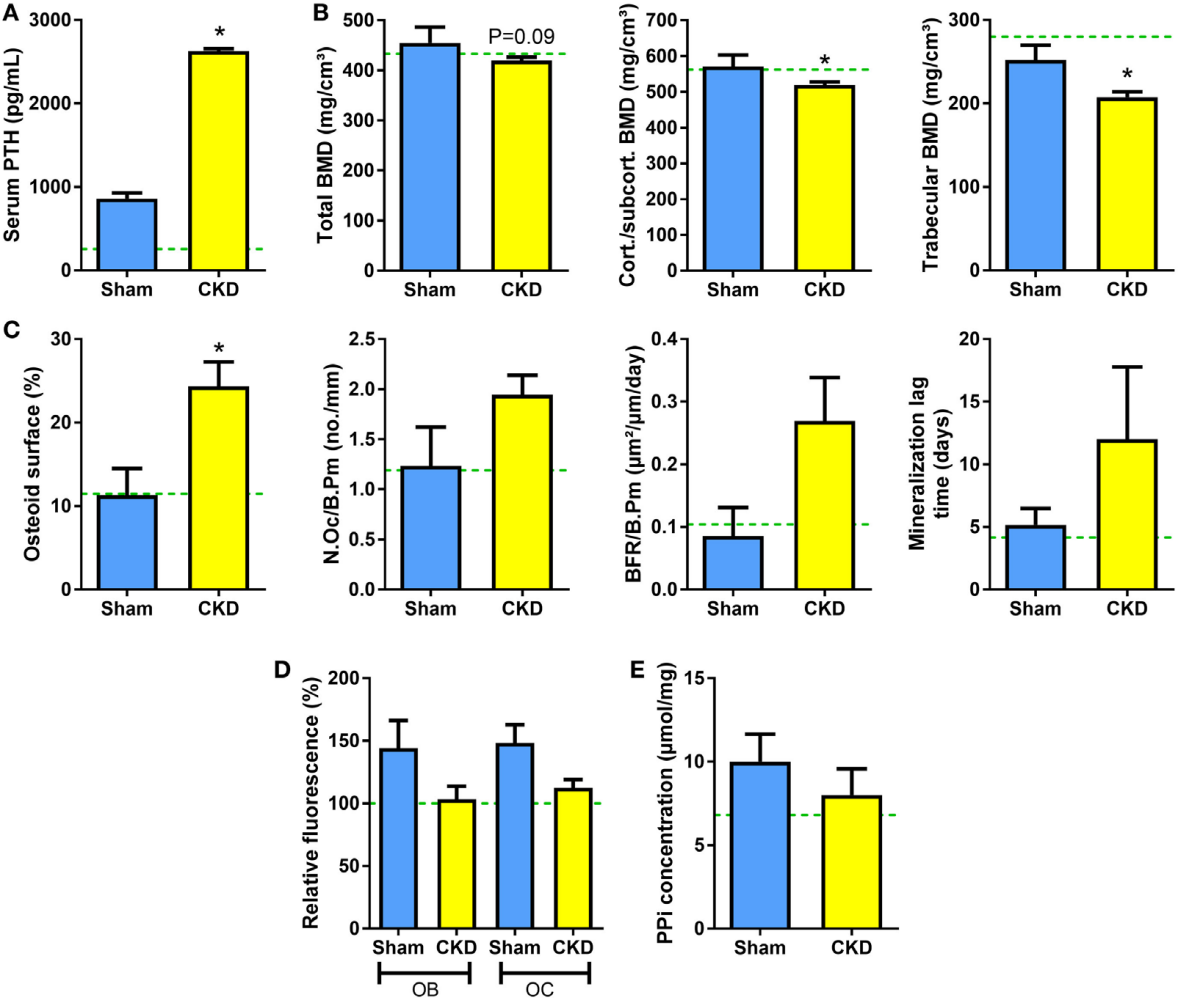
5/6-Nx induces bone loss in *Fgf23*/VDR compound mutant mice, but does not alter tissue nonspecific alkaline phosphatase (TNAP) activity or pyrophosphate concentration in bone. **(A)** Serum parathyroid hormone (PTH) (*n* = 13 each), **(B)** total, cortical/subcortical, and trabecular bone mineral density (BMD) of the L2 lumbar vertebra [4 Sham, 17 chronic kidney disease (CKD)], **(C)** osteoid surface (4 Sham, 14 CKD), number of osteoclasts (4 Sham, 14 CKD), bone formation rate (3 Sham, 10 CKD), and mineralization lag time (3 Sham, 10 CKD) in proximal tibial cancellous bone, **(D)** histochemical TNAP staining of undecalcified sections of proximal tibiae and quantification of relative fluorescence in osteoblasts (OB) and osteocytes (OC) (*n* = 3–5 each), and **(E)** pyrophosphate (PPi) concentration in extracts of femurs (*n* = 4–6 each) from 5-month-old male Sham and CKD *Fgf23*/VDR compound mutant mice on rescue diet, 8 weeks postsurgery. Data are mean ± SEM. Green dotted lines represent mean values found in wild-type (WT) Sham mice. The data in **(D)** were normalized to the WT Sham group. In **(A–C)**, * denotes *P* < 0.05 vs. Sham by *t*-test or Mann–Whitney *U*-test, as appropriate.

To test whether genetic ablation of *Fgf23* would rescue the suppression of TNAP activity and pyrophosphate accumulation in CKD mice, we performed 5/6-Nx in *Fgf23*^−/−^/VDR^Δ/Δ^ (*Fgf23*/VDR) compound mutants, which are characterized by concomitant *Fgf23* deficiency and lack of a functioning vitamin D receptor (VDR^Δ/Δ^). Single *Fgf23* knockout mice have a severe phenotype (35, 36), making it impossible studying 5/6-Nx-induced bone loss in these mice. However, the phenotype and early lethality of *Fgf23*^−/−^ mice can be rescued by ablation of the vitamin D signaling pathway (21). *Fgf23*/VDR compound mutants kept on rescue diet are healthy and can be studied until old ages (37). The rescue diet enriched with calcium, phosphate, and lactose is an elegant dietary tool to normalize blood calcium and PTH levels in VDR-ablated mice (23, 38). Employing this genetic loss-of-function model, we found that 5/6-Nx induced secondary hyperparathyroidism and bone loss in *Fgf23*/VDR mice, similar to WT mice (Figures 5A,B). In line with our previously reported finding that *Fgf23*/VDR mice are characterized by partial renal and skeletal PTH resistance, intact PTH levels were several-fold higher in Sham *Fgf23*/VDR mice than in Sham WT mice (Figure 5A). Nevertheless, 5/6-Nx still led to a ~3-fold upregulation of serum intact PTH in *Fgf23*/VDR mice (Figure 5A). Similar to WT mice, the CKD-induced bone loss in *Fgf23*/VDR mice was associated with increased osteoid surface (Figure 5C). However, in contrast to WT mice, osteoclast numbers and mineralization lag time in CKD *Fgf23*/VDR mice were not significantly different from Sham *Fgf23*/VDR mice, and showed, together with bone formation rate, only non-significant trends toward higher levels in CKD vs. Sham mice (Figure 5C). In agreement with the notion that the CKD-induced upregulation in osteoblastic and osteocytic Fgf23 secretion contributes to the mineralization defect in CKD mice, 5/6-Nx failed to significantly suppress TNAP activity in *Fgf23*/VDR mice (Figure 5D), and pyrophosphate concentrations remained unchanged in bones of CKD *Fgf23*/VDR mice (Figure 5E).

## Discussion

**Figure 6:**
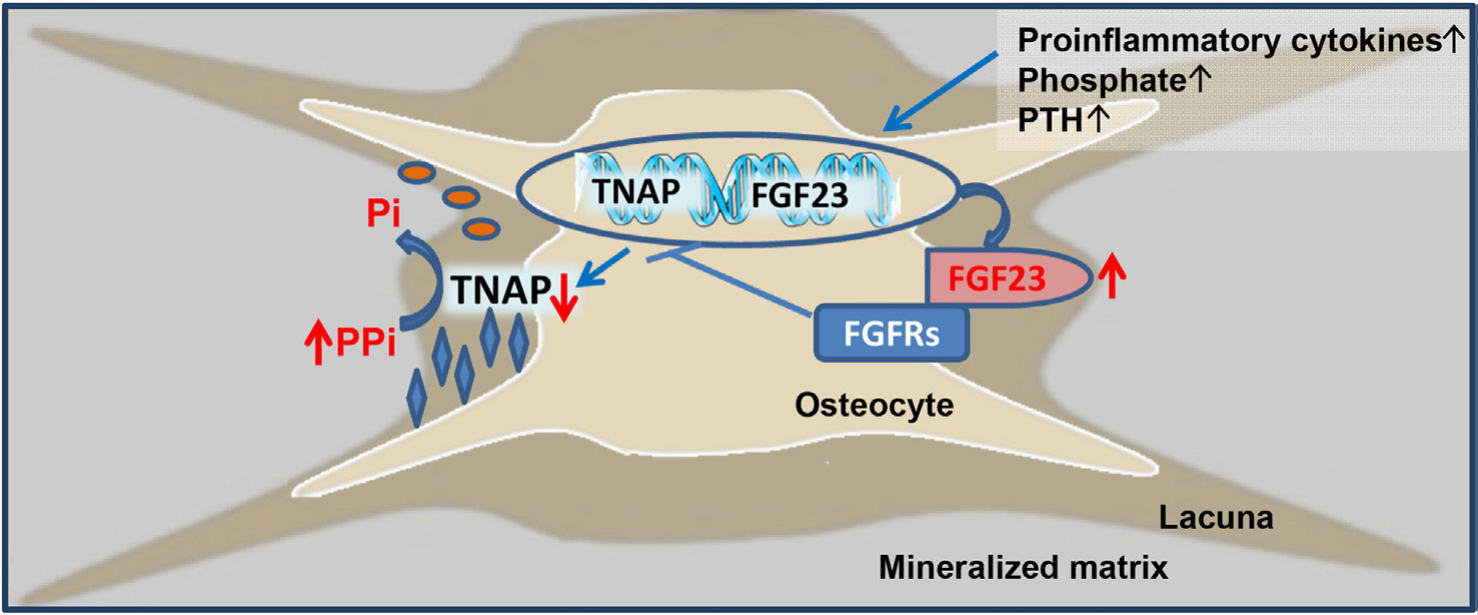
Proposed model of fibroblast growth factor-23 (FGF23)-induced suppression of tissue nonspecific alkaline phosphatase (TNAP) and accumulation of pyrophosphate in chronic kidney disease (CKD). Driven by increased circulating concentrations of pro-inflammatory cytokines, phosphate, parathyroid hormone (PTH), and other unknown factors, osteoblastic and osteocytic FGF23 secretion is increased in CKD. Excessive concentrations of FGF23 in the extracellular fluid surrounding osteocytes lead to FGF receptor (FGFR)-mediated, Klotho-independent suppression of *TNAP* transcription in a para-/autocrine manner. One of the main functions of TNAP in the mineralization process is the hydrolysis of the mineralization inhibitor pyrophosphate (PPi), thus providing inorganic phosphate (Pi) for mineralization. Hence, excessive FGF23 concentrations in the osteocyte canalicular network lead to accumulation of PPi and inhibition of bone mineralization in CKD.

The current study has shown that 5/6-Nx C57BL/6 mice on the phosphate-rich rescue diet develop bone loss and impaired bone mineralization within 8 weeks postsurgery. Furthermore, we found that the CKD-driven increase in osteoblastic and osteocytic Fgf23 secretion contributes to the mineralization defect in murine CKD-MDB by auto-/paracrine suppression of TNAP and subsequent accumulation of pyrophosphate in bone. Hence, our study has uncovered a novel mechanism involved in the pathogenesis of CKD-MBD, i.e., Fgf23-driven accumulation of the mineralization inhibitor pyrophosphate. This new paradigm is shown in Figure 6.

We previously showed that the Fgf23-induced auto-/paracrine suppression of TNAP in osteoblasts and osteocytes is caused by a Klotho-independent, mainly FGFR3-mediated signaling mechanism (18). The presence of transmembrane Klotho increases the affinity of FGFR1c to FGF23 by a factor of about 20 (39). Therefore, one of the key questions in this context is whether the concentrations of Fgf23 in the extracellular fluid surrounding osteoblasts/osteocytes is high enough for Klotho-independent FGFR signaling. The true concentration of Fgf23 within the osteocyte canalicular network is currently unknown. However, due to the fact that Fgf23 is secreted locally from bone cells, it is likely that the concentration is much higher within the canalicular network compared with the blood. In addition, differentiation of osteoblasts into osteocytes is associated with a profound upregulation of FGFR1 and 3 mRNA expression *in vivo* (20). Therefore, it is well conceivable that the Fgf23 concentration within the canalicular network is indeed sufficient to suppress TNAP transcription *via* a Klotho-independent, auto-/paracrine feedback mechanism. The situation is more complicated in osteoblasts. Osteoblasts are polar cells, and it is currently unknown whether FGF23 is secreted in a polar manner. In addition, it is unknown whether the membrane distribution of FGFRs is uniform or whether it is also organized in a polar fashion in osteoblasts. Therefore, it is difficult to estimate the concentrations of locally produced Fgf23 in the extracellular fluid surrounding osteoblasts, and to judge the possibilities for local feedback mechanisms. Nevertheless, our finding that the CKD-induced suppression of osteoblastic and osteocytic TNAP activity and accumulation of pyrophosphate in bone did not occur in *Fgf23*/VDR mutant mice lacking *Fgf23* lends additional support to the notion that the CKD-driven increase in Fgf23 secretion suppresses TNAP transcription in an auto-/paracrine manner.

Despite the increase in pyrophosphate concentrations in bone, 5/6-Nx did not increase the size of osteocyte lacunae in cortical bone in the current study. This finding is not unexpected for two reasons. First, we did not observe major increases in osteoid thickness in cancellous bone, making it unlikely to see major changes in the amount of osteoid in osteocyte lacunae, and, therefore, in lacunar size. Second, murine cortical bone does not show Haversian remodeling (33). Since 5/6-Nx was performed in adult mice, analysis of osteocyte lacunar size necessarily included mostly osteocytes formed prior to the onset of renal disease.

Total serum ALP activity was increased in CKD mice in our study. Similarly, bone-specific ALP is typically increased in patients with high turnover CKD-MBD (40). ALP activity in serum originates in about equal parts from bone and liver, with TNAP being the major isoenzyme found in blood (41). How can increased circulating ALP activity be reconciled with our finding that excessive Fgf23 secretion locally suppresses TNAP in bone cells? We do not have a conclusive answer to this question. However, the most likely explanation is that the number of osteoblasts and of osteoblast precursor cells is increased due to secondary hyperparathyroidism in CKD. Therefore, serum ALP activity may be mainly reflecting the number of osteoblasts and of osteoblast precursors rather than the ALP activity of individual bone cells. In line with this idea, we found a ~3-fold increase in osteoblast surface in WT CKD mice, relative to Sham controls.

Despite an approximately twofold increase in osteoclast numbers in proximal tibial cancellous bone, 24-h excretion of collagen crosslinks was reduced in CKD mice in our study. The plasma clearance of collagen crosslinks depends on kidney function (42). Therefore, serum collagen crosslink concentrations are not considered suitable markers of bone resorption in CKD patients (40). Under normal circumstances, urinary collagen crosslink excretion is the best estimate of whole body bone resorption activity in mice, because osteoclast numbers are often not indicative of osteoclast resorptive activity (33). Similarly, osteoblast surface and calcein-based bone formation rate were dissociated in the current study, underscoring the potential discrepancies between cell morphology and functional readouts in disease models. Whether, despite potential accumulation in serum, urinary collagen crosslink excretion accurately reflects bone resorption in CKD mice is not known. However, in a steady state, urinary excretion of collagen crosslinks should equal collagen breakdown in bone also in animals with compromized kidney function. Nevertheless, it remains unclear whether bone resorption in our CKD mouse model was increased as suggested by histomorphometry or decreased as suggested by urinary collagen crosslink excretion.

It is well known that circulating intact PTH is one of the main drivers of bone turnover in CKD patients. Hence, serum intact PTH is a useful biomarker to discriminate between low and high bone turnover in CKD patients (17), albeit its sensitivity to dissect high and low bone turnover disease is only about 65% (17, 40). What is actually causing the often observed impairment in bone mineralization in bone biopsies of CKD patients is not well known (1). One possibility is that secondary hyperparathyroidism *per se* leads to disturbed mineralization due to excessive stimulation of matrix synthesis and woven bone formation (1). Moreover, kidney-derived circulating Wnt inhibitors such as dickkopf-1 or sclerostin may inhibit bone formation and mineralization (43). In addition, FGF23 has been shown to directly suppress Wnt signaling in osteoblasts, and circulating uremic toxins and metabolic acidosis may interfere with normal osteoblast function and maturation (44–46). Our study has added an additional mechanism, namely the Fgf23-induced suppression of TNAP in osteoblasts and osteocytes. TNAP is essential for the initiation of bone mineralization by cleavage of pyrophosphate (19, 47). It is currently technically challenging to assess differences in local pyrophosphate concentration within the bone matrix. However, we hypothesize that increased pyrophosphate concentrations are at least a partial explanation for the profound increase in non-mineralizing osteoid seams in CKD mice in our study. This model may also help to explain the hungry bone syndrome in CKD patients after parathyroidectomy or kidney transplantation, because the decline in bony FGF23 secretion postsurgery will lift the FGF23-induced suppression of TNAP, leading to increased mineralization of previously unmineralized osteoid, and, hence, increased flux of calcium and phosphate into bone.

In conclusion, here we report a novel mechanism leading to impaired bone mineralization in CKD-MBD. We found that excessive CKD-driven Fgf23 secretion in osteoblasts and osteocytes is leading to auto-/paracrine suppression of TNAP and subsequent accumulation of the mineralization inhibitor pyrophosphate.

## Ethics Statement

All animal studies were approved by the Ethical Committee of the University of Veterinary Medicine, Vienna and by the Austrian Federal Ministry of Science and Research and were undertaken in strict accordance with prevailing guidelines for animal care (permit No. BMWF-68.205/0054-II/3b/2013). All efforts were made to minimize animal suffering.

## Author Contributions

OA and RE conceived and designed the experiments, analyzed the data, and wrote the manuscript; OA, CS, CB, and AP performed experiments.

## Conflict of Interest Statement

The authors declare that the research was conducted in the absence of any commercial or financial relationships that could be construed as a potential conflict of interest.
